# Unrelated cord blood transplantation for adult patients with acute myeloid leukemia: higher incidence of acute graft-versus-host disease and lower survival in male patients transplanted with female unrelated cord blood—a report from Eurocord, the Acute Leukemia Working Party, and the Cord Blood Committee of the Cellular Therapy and Immunobiology Working Party of the European Group for Blood and Marrow Transplantation

**DOI:** 10.1186/s13045-015-0207-4

**Published:** 2015-10-06

**Authors:** Frédéric Baron, Myriam Labopin, Annalisa Ruggeri, Mohamad Mohty, Guillermo Sanz, Noel Milpied, Andrea Bacigalupo, Alessandro Rambaldi, Francesca Bonifazi, Alberto Bosi, Jorge Sierra, Ibrahim Yakoub-Agha, Josep Maria Ribera Santasusana, Eliane Gluckman, Arnon Nagler

**Affiliations:** Department of Hematology, University of Liège, CHU Sart-Tilman, 4000 Liège, Belgium; EBMT Paris Office, Hospital Saint Antoine, Paris, France; Eurocord, Hospital Saint Louis, AP-HP, and IUH University Paris VII, Paris, France; AP-HP, Hématologie Clinique et Thérapie Cellulaire, Hôpital Saint-Antoine, Paris, France; Hospital Universitario La Fe - Servicio de Hematologia, Valencia, Spain; CHU Bordeaux - Hôpital Haut-leveque, Pessac, France; Department of Haematology II, Ospedale San Martino, Genova, Italy; Azienda Ospedaliera Papa Giovanni XXIII—Hematology and Bone Marrow Transplant Unit, Bergamo, Italy; Institute of Hematology and Medical, Oncology L and A Seràgnoli, S.Orsola-Malpighi Hospital, Bologna University, Bologna, Italy; BMT Unit Department of Hematology, Ospedale di Careggi, Firenze, Italy; Hematology Department, Hospital Santa Creu i Sant Pau, Barcelona, Spain; Hôpital HURIEZ - UAM allo-CSH - CHRU, Lille, France; ICO-Hospital Universitari Germans Trias i Pujol, Jose Carreras Research Institute, Barcelona, Spain; Eurocord, Hospital Saint Louis, AP-HP, and IUH University Paris VII, France Monacord, Centre Scientifique de Monaco, Monaco, Monaco; Division of Hematology and Bone Marrow Transplantation, The Chaim Sheba Medical Center, Tel-Hashomer, Ramat-Gan, Israel

**Keywords:** Unrelated cord blood, Female, Male, AML, GVHD, Transplantation

## Abstract

**Background:**

In the setting of allogeneic human leukocyte antigen (HLA)-matched bone marrow transplantation, transplanting male patients with grafts from female donors has been associated with a higher incidence of graft-versus-host disease (GVHD) and of nonrelapse mortality (NRM). The aim of the current analysis was to compare transplantation outcomes in male patients given female unrelated cord blood (UCB) versus other gender combinations.

**Patients and methods:**

Data from 552 consecutive patients with acute myeloid leukemia (AML) given a single UCB transplantation between 2000 and 2014 were included.

**Results:**

In comparison with other gender combination, male patients given female UCB (*n* = 131) had a trend for a higher incidence of grades II–IV acute GVHD (33 versus 25 %, *P* = 0.08), a trend for a higher incidence of NRM (41 versus 33 %, *P* = 0.06), and a lower leukemia-free (LFS, 30 versus 41 %, *P* = 0.01) and overall survival (OS, 33 versus 45 %, *P* = 0.008). In multivariate analyses, taking into consideration all patients for which data on HLA-matching and cell dose transplanted were fully available (*n* = 363), male patients transplanted with a female UCB had a trend for a higher incidence of grade III–IV acute GVHD (hazard ratio (HR) = 2.0, *P* = 0.06), a trend for a higher NRM (HR = 1.5, *P* = 0.06), and a worse LFS (HR = 1.4, *P* = 0.04) and OS (HR = 1.3, *P* = 0.06).

**Conclusions:**

Our data suggest that male patients transplanted with female UCB might have higher risk of acute GVHD and of NRM leading to worse LFS and OS. These results should be confirmed in other large cohorts of patients before used for determining the choice of an UCB unit.

**Electronic supplementary material:**

The online version of this article (doi:10.1186/s13045-015-0207-4) contains supplementary material, which is available to authorized users.

## Background

Allogeneic hematopoietic stem cell transplantation from human leukocyte antigen (HLA)-identical sibling is the treatment of choice for many patients with acute myeloid leukemia (AML) [1, 2]. For patients who lack a suitable HLA-identical sibling, unrelated cord blood transplantation (UCBT) and HLA-haploidentical transplantation have emerged as an adequate alternative to HLA-matched unrelated bone marrow/peripheral blood stem cell transplantation, particularly for patients at high risk of rapid disease relapse who urgently need a transplantation [3–7].

Despite major improvements in the field in the last decades [8], nonrelapse mortality (NRM) has remained the main cause of failure of UCBT for AML [4]. In the setting of allogeneic HLA-matched bone marrow or peripheral blood stem cell transplantation, transplanting male patients with grafts from female donors has been associated with a higher incidence of graft-versus-host disease (GVHD) leading to higher NRM and a lower overall survival (OS) [9, 10]. This is due to recognition by female donor immune cells of minor histocompatibility antigens (HA) encoded by genes on the recipient Y chromosome that are polymorphic to their X chromosome homologue [11–15]. These HA, termed H-Y antigens, are highly immunogenic and are expressed throughout the body [15].

Donor naïve T cells recognizing major or minor histocompatibility antigens expressed on normal host tissues are largely involved in GVHD pathogenesis [16–18]. However, recent findings suggest that effector memory T cells might also play a role in GVHD pathogenesis in humans [19].

Despite it has been considered that cord blood T cells were mostly naïve and dedicated to the development of tolerance, recent findings evidence the presence of CD4+ T cells with effector memory phenotype and function in cord blood, representing 1–3 % of CD4+ T cells and behaving as inflammatory cells with a mixed Th1- and Th2-like function upon activation [20]. Further, a recent study demonstrated that the presence of HLA-allele mismatch(es) increased NRM after UCBT [21], demonstrating that alloreactivity after UCBT was not restricted to HLA-antigen mismatches between recipients and UCB but suggesting a role also for minor HA in GVHD pathogenesis following UCBT. These findings prompted us to assess whether transplanting male AML patients with female UCB has an impact on GVHD and UCBT outcomes.

## Results

### Patient, disease, and transplant characteristics

Data from 552 consecutive patients with AML given a single unit UCBT between 2000 and 2014 were included. Their characteristics are described in Table 1. Briefly, 131 patients were male patients given female UCB, 119 patients were male patients receiving male UCB, and 302 were female patients. In comparison to other patients, male patients given female UCB were less likely to have high-risk cytogenetic or secondary leukemia (39 versus 62 %, *P* = 0.005), were less often transplanted following a reduced-intensity conditioning (24 versus 35 %, *P* = 0.03), received more often cyclosporine A alone as GVHD prophylaxis (39 versus 33 %, *P* = 0.001), and received less total nucleated cells (2.5 versus 2.8 × 10^7^/kg, *P* = 0.01). The other characteristics were well-balanced between the two groups of patients.

**Table 1:** Patient, transplant characteristics and GVHD

	Other gender combination (*n* = 421)	Female UCB to male recipient (*n* = 131)	Pvalue^a^
Median patient age, years (range)	43 (18–70)	42 (18–67)	0.48
Median year of SCT, years (range)	2009 (2000–2013)	2009 (2000–2013)	0.24
Recipient M donor F, # (%)	0 (0)	131 (100)	/
Recipient M donor M, # (%)	119 (28)	0 (0)	
Recipient F donor F, # (%)	157 (37)	0 (0)	
Recipient F donor M, # (%)	145 (34)	0 (0)	
Median time from diagn to SCT, days	210	231	0.48
Secondary AML, # (%)	72 (17)	18 (14)	0.36
Status at transplantation, # (%)			
CR1	233 (55)	59 (45)	0.12
CR2+	92 (22)	36 (27)	
Advanced	96 (23)	36 (27)	
Cytogenetics, # (%)			
Good risk^b^	17 (9)	14 (21)	0.005
Intermediate risk^c^	56 (29)	26 (39)	
High risk^d^	47 (25)	8 (12)	
Not reported/failed	229	65	
Secondary AML	72 (37)	18 (27)	
Recipient CMV-seronegative, # (%)	100 (28)	26 (27)	0.83
Conditioning regimen, # (%)			
Myeloablative	271 (65)	94 (76)	0.03
Reduced-intensity	145 (35)	30 (24)	
ATG, # (%)	232 (61)	65 (56)	
TNC at infusion ×10^7^/kg			
Median (range)	2.8 (0.33–40.3)	2.5 (0.4–6.10)	0.01
Missing data (# of patients)	71	19	
Number of HLA disparities, # (%)			
0–1 mismatch	121 (36)	35 (33)	
2–3 mismatches	213 (64)	70 (67)	0.59
Missing data	87	26	
Postgrafting immunosuppression, # (%)			
CSP alone	140 (33)	51 (39)	0.19
CSP + MMF	215 (51)	55 (42)	
Other/missing	66 (16)	25 (19)	
Acute GVHD, # (%)			
Grades I–IV	171 (43)	59 (49)	0.28
Grades II–IV	99 (25)	40 (33)	0.08
Grades III–IV	43 (11)	18 (15)	0.23
CI chronic GVHD @ 2 years, %	28	21	0.44
Death from GVHD, # (%)	29 (7)	10 (8)	0.77

*M* male, *F* female, *SCT* stem cell transplantation, *diagn* diagnosis, *#* number of patients, *tacro* tacrolimus, *CSP* cyclosporine A, *Mtx* methotrexate, *MMF* mycophenolate mofetil

^**a**^Calculated with *χ* ^2^ statistics for categorical variables and Mann-Whitney test for continuous variables

^b^Defined as t(8;21), t(15;17), inv or del (16), acute promyelocyticleukemia, or these abnormalities only or combined with others

^c^Defined as all cytogenetics not belonging to the good or high risk (including trisomias)

^d^Defined as 11q23 abnormalities, complex caryotype, and abnormalities of chromosomes 5 and 7

### Engraftment

**Fig. 1:**
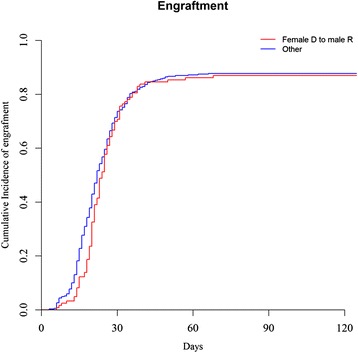
Cumulative incidence of neutrophil engraftment in male patients given female UCB (*n* = 131) versus in other gender combinations (*n* = 421)

Overall, cumulative incidence of neutrophil engraftment at day 100 was similar in male patients given female UCB (87 % (95 % confidence interval (CI), 79.5–91.9)) versus in other gender combinations (87.7 % (95 % CI, 84–90.6), *P* = 0.31). Interestingly, median times for reaching 0.5 × 10^9^/L neutrophils were 23 days (range, 6–68 days) in male patients given female UCB versus 21 days (range, 3–66 days, *P* = 0.02) in other gender combinations (Fig. 1). This could be possibly due to the fact that male patients given female UCB received less total nucleated cell counts (TNC), or this could be due to the fact that, according previous observations, male CB units include more CD34+ cells and more colony-forming unit than female ones [22].

Given that cytotoxic T cells (CTLs) directed against antigens coded by the Y chromosome have been associated with graft rejection in female patients transplanted with bone marrows from male HLA-identical siblings [23], we also compared engraftment kinetics in female patients transplanted with male UCB (*n* = 145) versus other patients (*n* = 407). Overall, the 100-day cumulative incidence of neutrophil engraftment was similar in female patients given male UCB (88.3 % (95 % CI, 81.5–92.7)) and in other gender combinations (87.3 % (95 % CI, 83.5–90.3), *P* = 0. 68). Median times for reaching 0.5 × 10^9^/L neutrophils were 21 days (6–49) in female patients transplanted with male UCB versus 21 days (3–66 days, *P* = 0.71) in other patients.

### GVHD

Male patients transplanted with female UCB had a trend for a higher incidence of grades II–IV acute GVHD (33 versus 25 %, *P* = 0.08) than other patients, while rates of grades III–IV acute GVHD were 15 versus 11 % (*P* = 0.2) in male patients given female UCB and in other patients, respectively (Table 2). In multivariate analyses, male patients transplanted with female UCB had significantly higher incidences of grades II–IV (hazard ratio (HR) 1.7, 95 % CI 1.0–2.7; *P* = 0.04) and grades III–IV (HR 1.9, 95 % CI 1.0–3.6; *P* = 0.04) acute GVHD than other patients. Restricting the multivariate analyses to patients for which data on TNC transplanted and HLA compatibility were available (*n* = 363, including 87 male patients given female UCB), there was still a trend for a higher incidence of grades II–IV (HR 1.7, 95 % CI 1.0–3.0; *P* = 0.07) and grades III–IV (HR 2.0, 95 % CI 1.0–3.9; *P* = 0.06) acute GVHD in male patients given female UCB, versus other gender combinations (Table 3). Restricting the analyses to male recipients (*n* = 250), those given female UCB had an incidence of grades II–IV acute GVHD of 33 %, compared with 27 % (*P* = 0.32) for those transplanted with male UCB. Finally, the incidence of grades II–IV acute GVHD was 21 % in female recipients given female UCB (*P* = 0.02 in comparison to male patients given female UCB).

**Table 2:** Two-year outcomes according to gender combination

	RI	NRM	LFS	OS	cGVHD
Male to male	22.8 % (15.4–31.1)	36.6 % (28.1–45.1)	40.7 % (31.3–50)	42.4 % (32.8–52)	15.7 % (8.3–25.2)
Female to male	29.2 % (21.1–37.8)	40.8 % (32.3–49.2)	29.9 % (21.3–38.6)	33 % (24.1–41.9)	10.1 % (4.7–18)
Male to female	29 % (21–37.5)	35.1 % (26.7–43.6)	35.9 % (27.1–44.7)	40.9 % (32.1–49.8)	13 % (6.9–21.1)
Female to female	26.3 % (18.9–34.2)	28.4 % (20.4–36.9)	45.3 % (36.5–54.1)	51 % (42.3–59.7)	11.6 % (6.1–19)
*P*	0.8096	0.098598	0.040965	0.030146	0.83169

**Table 3:** Multivariate analyses in patients for which data on total nucleated cell counts (TNC) and HLA compatibility were available (*n* = 363, including 87 male patients given female UCB) (significant factors are in italic)

Logistic regression		*P*	OR	95 % CI	
AGVH II+	Female to male	0.07	1.7	1.0	3.0
	Age (years)	0.60	1.0	1.0	1.0
	Status at Tx				
	CR1 versus CR2	0.07	0.6	0.3	1.0
	*CR1 versus advanced*	*0.04*	*0.5*	*0.2*	*1.0*
	*ATG*	*0.02*	*0.5*	*0.3*	*0.9*
	RIC	0.20	1.5	0.8	2.9
	Sec AML	0.59	0.8	0.4	1.7
	Infused cells >2.7 (median)	0.25	0.7	0.5	1.2
	Nr mism >1	0.70	1.1	0.7	1.8
AGVH III–IV	Female to male	0.06	2.0	1.0	3.9
	Age (years)	0.90	1.0	1.0	1.0
	Status at Tx				
	CR1 versus CR2	0.38	0.7	0.3	1.5
	*CR1 versus advanced*	*0.05*	*0.4*	*0.1*	*1.0*
	*ATG*	*0.03*	*0.5*	*0.2*	*0.9*
	RIC	0.80	1.1	0.5	2.6
	Sec AML	0.56	1.3	0.6	3.0
	Infused cells >2.7 (median)	0.99	1.0	0.5	1.9
	Nr mism >1	0.84	0.9	0.5	1.8
Cox models		*P*	HR	95 % CI	
NRM	Female to male	0.06	1.5	1.0	2.2
	Age (years)	0.21	1.0	1.0	1.0
	Status at Tx				
	*CR1 versus CR2*	*0.01*	*0.5*	*0.3*	*0.8*
	CR1 versus advanced	0.62	0.9	0.5	1.5
	*ATG*	*0.04*	*1.5*	*1.0*	*2.4*
	RIC	0.09	0.6	0.4	1.1
	Sec AML	0.06	1.6	1.0	2.4
	Infused cells >2.7 (median)	0.18	0.8	0.5	1.1
	Nr mism >1	0.18	0.8	0.5	1.1
RI	Female to male	0.40	1.2	0.8	1.9
	Age (years)	*0.03*	*1.0*	*1.0*	*1.0*
	Status at Tx				
	*CR1 versus CR2*	*<10–4*	*0.2*	*0.1*	*0.4*
	*CR1 versus advanced*	*<10–4*	*0.3*	*0.2*	*0.5*
	ATG	0.88	1.0	0.6	1.5
	*RIC*	*0.04*	*1.7*	*1.0*	*2.9*
	*sec AML*	*0.00*	*0.2*	*0.1*	*0.4*
	Infused cells > 2.7 (median)	0.06	0.7	0.5	1.0
	Nr mism > 1	0.64	0.9	0.6	1.4
LFS	*Female to male*	*0.04*	*1.4*	*1.0*	*1.9*
	*Age (years)*	*0.03*	*1.0*	*1.0*	*1.0*
	*Status at Tx*				
	*CR1 versus CR2*	*<10–4*	*0.4*	*0.3*	*0.5*
	*CR1 versus advanced*	*0.01*	*0.6*	*0.4*	*0.9*
	ATG	0.14	1.3	0.9	1.7
	RIC	0.90	1.0	0.7	1.5
	Sec AML	0.17	0.8	0.5	1.1
	*Infused cells >2.7 (median)*	*0.04*	*0.7*	*0.6*	*1.0*
	Nr mism >1	0.23	0.8	0.6	1.1
OS	Female to male	0.06	1.3	1.0	1.8
	*Age (years)*	*0.01*	*1.0*	*1.0*	*1.0*
	*Status at Tx*				
	*CR1 versus CR2*	*<10–4*	*0.4*	*0.3*	*0.5*
	*CR1 versus advanced*	*0.01*	*0.6*	*0.4*	*0.9*
	ATG	0.23	1.2	0.9	1.7
	RIC	0.35	0.8	0.6	1.2
	Sec AML	0.35	0.8	0.6	1.2
	*Infused cells >2.7 (median)*	*0.05*	*0.8*	*0.6*	*1.0*
	Nr mism >1	0.40	0.9	0.7	1.2

*HR* hazard ratio, *CI* confidence interval, *P P* value, *AGVHD II+* grades II–IV acute GVHD, *AGVHD III–IV* grades III–IV acute GVHD, *cGVHD* cumulative incidence of chronic graft-versus-host disease, *NRM* cumulative incidence of nonrelapse mortality, *RI* cumulative incidence of relapse, *LFS* leukemia-free survival, *OS* overall survival, *Tx* transplantation, *CR* complete remission, *advanced* not in complete remission, *ATG* anti-thymocyte globulin, *RIC* reduced-intensity conditioning, *sec AML* secondary acute myeloid leukemia

Interestingly, 2-year incidence of chronic GVHD tended to be lower in male patients given female UCB in univariate analysis (16 versus 25 %, *P* = 0.11) (Table 2), while in multivariate analysis, the incidence of chronic GVHD was not different according to gender combination (HR = 0.8, 95 % CI 0.4–1.4; *P* = 0.4).

### Relapse and NRM

**Fig. 2:**
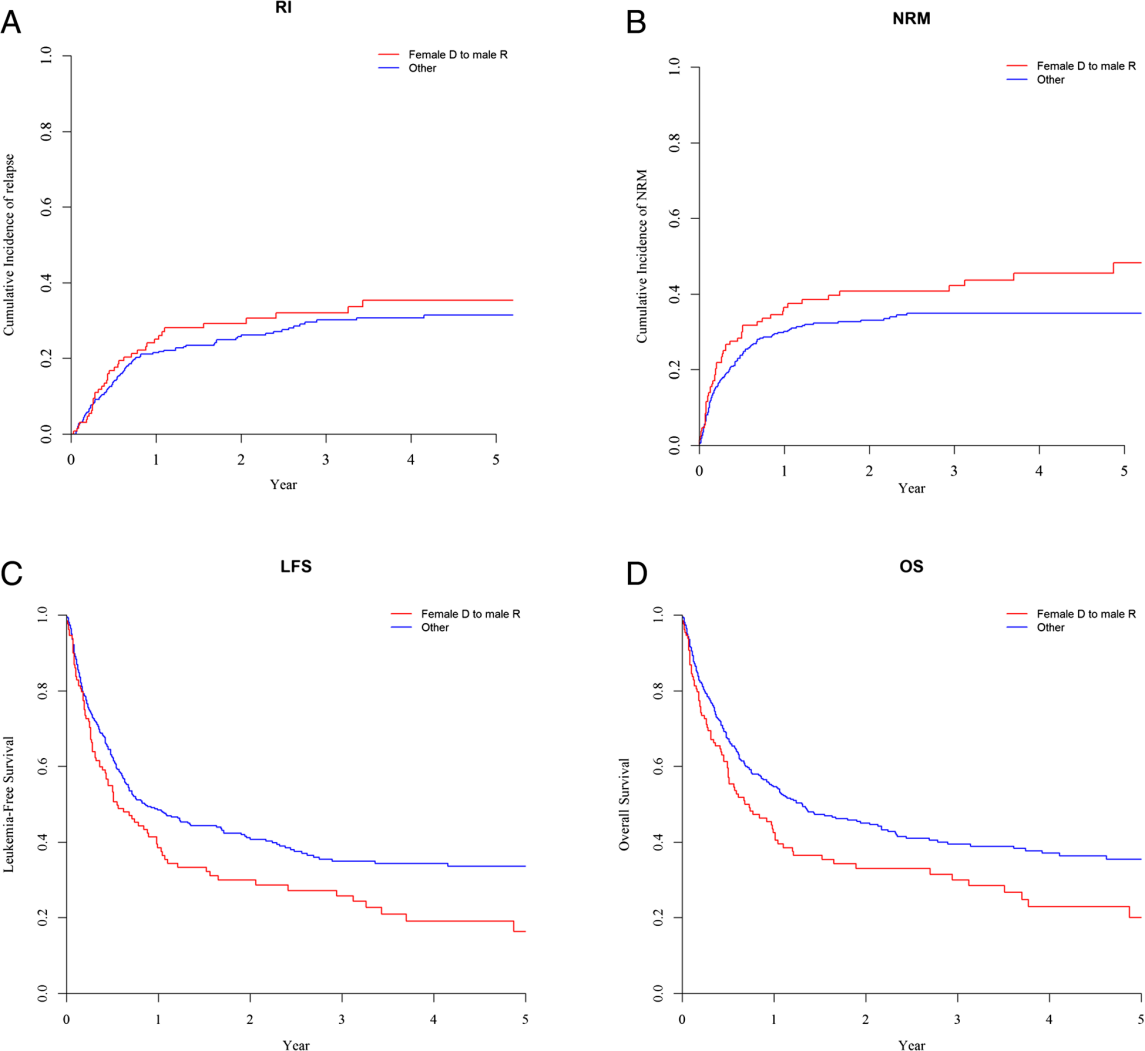
Relapse incidence (**a**), NRM (**b**), LFS (**c**), and overall survival (**d**) in male patients given female URD (*n* = 131) versus other gender combinations (*n* = 421)

**Fig. 3:**
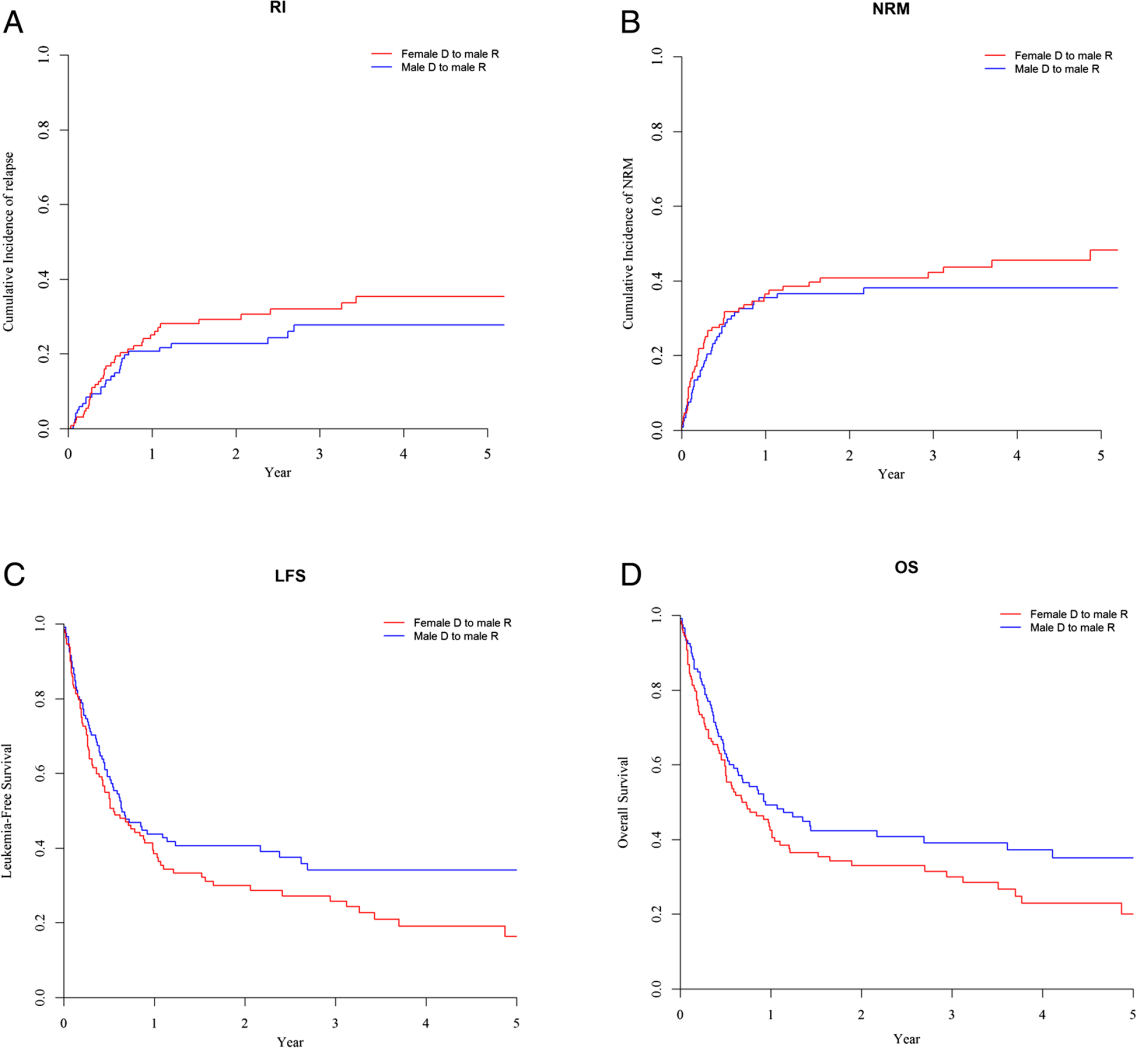
Relapse incidence (**a**), NRM (**b**), LFS (**c**), and overall survival (**d**) in male patients given female URD (*n* = 131) versus in male patients given male UCB (*n* = 119)

The 2-year incidence of relapse was similar in male patients given female UCB (29.2 %) and in other patients (26.2 %, *P* = 0.4) (Fig. 2). The figures were 22.8 % in male patients given male UCB (*P* = 0.35 in comparison to male patients given female UCB) (Fig. 3) and 26.3 % in female patients given female UCB (Table 2). In multivariate analyses including data from all patients, male patients given female UCB had a similar incidence of relapse than other gender combinations (HR = 1.4, 95 % CI 0.9–2.1; *P* = 0.13). Similar observations were made when the analyses were restricted to patients for whom data on TNC and HLA compatibility were available (HR = 1.2, 95 % CI 0.8–1.9; *P* = 0.4) (Table 3). Factors associated with increased relapse incidence in multivariate analysis included older recipient age (*P* = 0.03), CR2 or advanced disease (*P* < 0.001) versus CR1, reduced-intensity conditioning (RIC) (*P* = 0.04), and secondary AML (*P* < 0.001).

Two-year incidences of NRM were 40.8 versus 33.1 % (*P* = 0.06), respectively, in male patients given female UCB versus in other gender combinations (Fig. 2). The figures were 36.6 % in male patients given male UCB (*P* = 0.41 in comparison to male patients given female UCB) (Fig. 3) and 28.4 % in female patients given female UCB (Table 2). In multivariate analyses including data from all patients, male patients given female UCB had a significantly higher incidence of NRM than other patients (HR = 1.4, 95 % CI 1.0–2.0; *P* = 0.04). Restricting the analyses to patients for which data on TNC and HLA compatibility were available, there was still a trend for higher NRM in male patients transplanted with female UCB (HR = 1.5, 95 % CI 1.0–2.2; *P* = 0.06) (Table 3). Other factors associated with higher NRM in multivariate analyses included CR2 versus CR1 (*P* = 0.01) and the use of anti*-*thymocyte globulin (ATG) (*P* = 0.04).

### Overall and leukemia-free survival

Two-year lower leukemia-free survivals (LFS) were 29.9 versus 40.7 % (*P* = 0.01), respectively, in male patients given female UCB versus in other patients (Fig. 2). The figures were 40.7 % in male patients given male UCB (*P* = 0.11 in comparison to male patients given female UCB) (Fig. 3) and 45.3 % in female patients given female UCB (Table 2). In multivariate analyses including data from all patients, male patients given female UCB had a significantly worse LFS than other gender combinations (HR = 1.4, 95 % CI 1.1–1.8; *P* = 0.01). Restricting the analyses to patients for which data on TNC and HLA compatibility were available, LFS remained significantly worse in male patients transplanted with female UCB (HR = 1.4, 95 % CI 1.0–1.9; *P* = 0.04) (Table 3). Other factors associated with worse LFS in multivariate analysis included older age (*P* = 0.03), CR2 (*P* < 0.001) or advanced disease (*P* = 0.01) versus CR1, and low number of TNC infused (*P* = 0.04).

Two-year OS were 33 versus 45 % (*P* = 0.008), respectively, in male patients given female UCB versus in other gender combinations (Fig. 2). The figures were 42.4 % in male patients given male UCB (*P* = 0.10) in comparison to male patients given female UCB) (Fig. 3) and 51 % in female patients given female UCB (Table 2). In multivariate analyses including data from all patients, male patients given female UCB had a significantly worse OS than other gender combinations (HR = 1.4, 95 % CI 1.0–1.8; *P* = 0.02). Similar qualitative observations were made when restricting the analyses to patients for which data on TNC and HLA compatibility were available (HR = 1.3, 95 % CI 1.0–1.8; *P* = 0.06) (Table 3). Other factors associated with worse OS in multivariate analysis included older age (*P* = 0.01), CR2 (*P* < 0.001) or advanced disease (*P* = 0.01) versus CR1, and low number of TNC infused (*P* = 0.05). Causes of death were comparable in male patients transplanted with female UCB and in other gender combination. Specifically, main causes of death were disease progression (24 % of transplanted patients), infection (23 % of transplanted patients), and GVHD (8 % of transplanted patients) in male patients transplanted with female UCB versus disease progression (18 % of transplanted patients), infection (17 % of transplanted patients), and GVHD (7 % of transplanted patients) in other gender combinations.

### Graft-versus-leukemia effects of chronic GVHD?

**Fig. 4:**
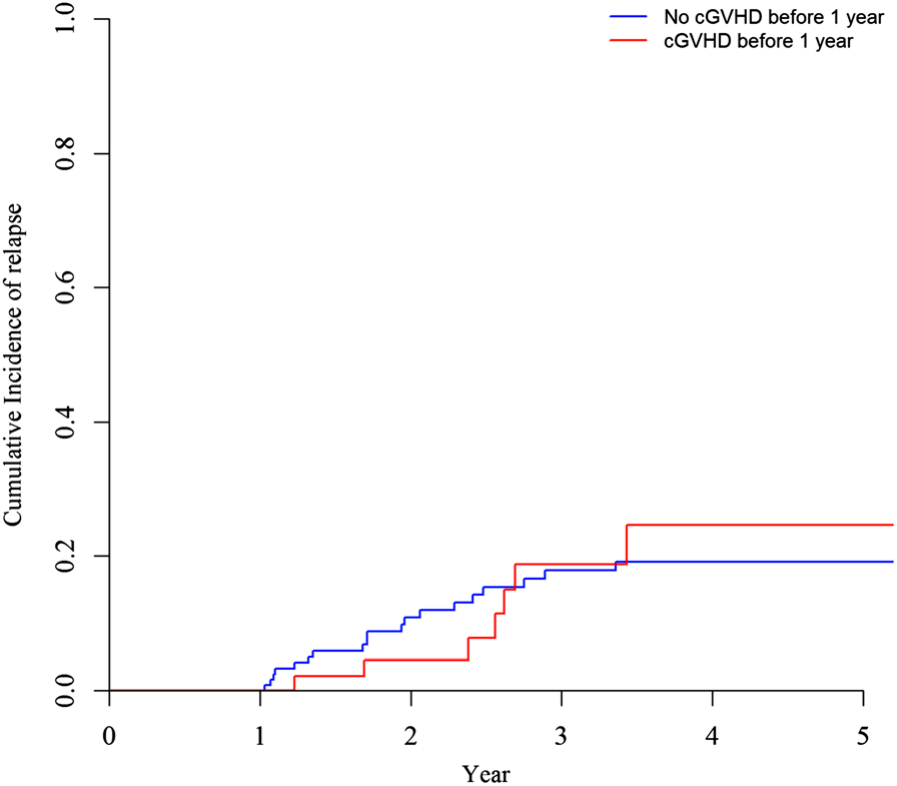
Relapse incidence in a landmark analysis selecting patients alive without relapse at 1 year after transplantation (*n* = 179) and considering chronic GVHD during the first year after transplantation (present in 54 patients)

Previous studies in the bone marrow or peripheral blood setting have demonstrated a strong link between chronic GVHD occurrence and a lower risk of relapse [24–27]. Since we did not observe a lower risk of relapse in male patients given female UCB, we assessed whether chronic GVHD was the driver of graft-versus-leukemia effects after UCBT. We first performed a landmark analysis selecting patients alive without relapse at 1-year post-transplant and considering chronic GVHD occurring the first year post-transplant (*n* = 179). As shown in the Fig. 4, 2-year cumulative incidence of relapse was 10.8 % (95 % CI 5.9–17.5 %) in patients without chronic GVHD before 1 year (*n* = 125), versus 4.5 % (95 % CI 0.8–13.7 %) in patients with chronic GVHD before 1 year (*n* = 54) (*P* = 0.9). We confirmed the absence of statistically significant association between chronic GVHD and graft-versus-leukemia effects in a multivariate Cox model that showed that occurrence of chronic GVHD (assessed as a time-dependent covariate) was not associated with a lower risk of relapse (HR = 1.2, 95 % CI 0.7–2.1).

## Discussion

Despite cord blood T cells are more tolerant than adult T cells [28, 29], a recent study demonstrated that the presence of HLA-allele mismatch(es) increased NRM after UCBT [21], evidencing that alloreactivity after UCBT was not restricted to HLA-antigen mismatches between recipients and UCB and suggesting a possible role for minor histocompatibility antigens in GVHD pathogenesis following UCBT. These findings prompted us to assess whether alloreactivity against H-Y antigens, a class of well-known highly immunogenic minor HA which are expressed throughout the body [15], played a role in the UCBT setting.

Main observations were that male patients given female UCB had a higher incidence of acute GVHD, leading to increased NRM and worse LFS and OS. These observations are in line with what has been observed in the setting of HLA-identical bone marrow or peripheral blood stem cell (PBSC) transplantation [9]. This suggests that even in the presence of HLA-mismatches, donor cord blood T cells are able to react against H-Y antigens. However, these results are in contrast to those reported by Konuma et al. who observed no impact of sex mismatch on acute GVHD, LFS, nor OS in a cohort of 191 patients who received a single unit UCBT as treatment for various malignancies [30].

Interestingly, in contrast to what was observed in HLA-identical bone marrow or PBSC recipients [9, 31, 32], male recipients of female UCB were not exposed to a higher incidence of chronic GVHD. The reason for this apparent discrepancy is unclear and might be related to differences in the biology of cord blood versus bone marrow transplantation.

Despite having a higher incidence of acute GVHD, male recipients of female UCB were not protected from relapse. While occurrence of acute and/or chronic GVHD has been associated with a lower risk of relapse in AML patients in the setting of HLA-matched bone marrow or peripheral blood stem cells transplantation [26, 33, 34], the impact of GVHD on transplantation outcomes in the UCBT setting remains to be investigated. Our current data suggest that transplanting male patients with female UCB is not associated with increased graft-versus-leukemia effects, in contrast to what has been observed after HLA-identical bone marrow transplantation [9]. These results mirror those observed in a study assessing the impact of HLA-allele mismatches in the UCBT setting where increasing mismatching correlated with acute GVHD and nonrelapse mortality but not with a protection from relapse [21]. These results are also in line with those reported by Konuma et al. who observed no impact of sex mismatch on relapse incidence after single unit UCBT [30].

The current study also confirmed a detrimental impact of ATG on NRM as recently reported in a study including data from patents given UCB after myeloablative conditioning [35], probably due to the negative impact of ATG on immune recovery after allogeneic stem cell transplantation [36]. However, ATG had no impact on relapse incidence, in agreement with recent observations in the peripheral blood stem cell setting [37–39]. Further, as previously observed in the UCB setting [4, 40], older age was associated with worse LFS and OS. Interestingly, while patients given RIC had a higher incidence of relapse, this did not translate to worse LFS or OS due to a trend to lower NRM in patients given RIC (*P* = 0.09), as previously observed in the setting of UCBT as treatment for ALL [40]. In addition, confirming previous observations, low TNC infused correlated to worse OS [29]. Finally, as expected, advanced disease status at transplantation had a negative impact of on all transplantation outcomes, while patients with secondary (versus primary) AML had a higher risk of relapse in contrast to recent observations in the PBSC transplantation setting [41].

There are some limitation in our study including its design (retrospective registry survey) and the surprising relative imbalance between the two groups. We tried to address these issues by performing multivariate analyses. Nevertheless, current results should be taken with some caution and should be confirmed in other large cohort of patients before recommendations can be made regarding the choice of the gender of the UCB in male patients with AML. Further, translational research looking at the presence (or absence) of CTLs [23] or antibodies [14] directed against antigens coded by the Y chromosome in male patients transplanted with female UCT are needed.

## Conclusions

In summary, our data suggest that male AML patients transplanted with female UCB might have higher risk of acute GVHD and of NRM leading to worse LFS and OS. These results should be confirmed in other large cohorts of patients before used for determining the choice of an UCB unit.

## Patients and methods

### Data Collection

This survey is a retrospective study performed by the Acute Leukemia Working Party (ALWP) of the European Group for Blood and Marrow Transplantation (EBMT) group and by Eurocord. EBMT registry is a voluntary working group of more than 500 transplant centers, participants of which are required once a year to report all consecutive stem cell transplantations and follow-up. Eurocord collects data on UCBT performed in >50 countries worldwide and >500 transplant centers, mainly EBMT centers. Population selection criteria included adult recipients, primary or secondary AML, first allogeneic stem cell transplantation, and single-unit UCBT performed from 2000 to 2014. Grading of acute and chronic GVHD was performed using established criteria [42]. HLA compatibility was based on low-resolution typing for HLA-A and HLA-B and high-resolution typing for HLA-DRB1. For the purpose of this study, all necessary data were prospectively collected according to EBMT and Eurocord guidelines.

### Ethics

The scientific boards of the ALWP of EBMT and of Eurocord approved this study.

### Statistical analyses

Data from all patients meeting the inclusion/exclusion criteria were included in the analyses(additional file). Start time was date of transplant for all endpoints. Neutrophil engraftment was defined as first of three consecutive days with a neutrophil count of at least 0.5 × 10^9^/L, while platelet engraftment was defined as the first of seven consecutive days of an unsupported platelet count of at least 20 × 10^9^/L.

To evaluate the relapse incidence, patients dying either from direct toxicity of the procedure or from any other cause not related to leukemia were censored. NRM was defined as death while in CR. Patients were censored at the time of relapse or of the last follow-up. Cumulative incidence functions (CIF) were used for relapse incidence and NRM in a competing risk setting, since death and relapse were competing together.

For estimating the cumulative incidence of chronic GVHD, death was considered as a competing event. OS and leukemia-free survival (LFS) were estimated using the Kaplan-Meier estimates. Univariate analyses were done using Gray’s test for CIF and log rank test for OS and LFS. Multivariate analyses adjusted for differences between groups were performed using Cox proportional hazards regression models for OS, LFS, relapse incidence, and NRM and using multivariate logistic regression for acute GVHD. The impact of chronic GVHD on the risk of relapse was assessed by performing a landmark analysis 1 year after transplantation and in a multivariate time-dependent Cox model in which chronic GVHD was modeled as a time-dependent covariate. All tests were two sided. The type I error rate was fixed at 0.05 for determination of factors associated with time to event outcomes. Statistical analyses were performed with SPSS 19 (SPSS Inc, Chicago, IL) and R 2.13.2 (R Development Core Team, Vienna, Austria) software packages.

## Additional file

Additional file 1:
**List of institutions reporting data in this study.**
