# Supplementary material for: Unrelated cord blood transplantation for adult patients with acute myeloid leukemia: higher incidence of acute graft-versus-host disease and lower survival in male patients transplanted with female unrelated cord blood—a report from Eurocord, the Acute Leukemia Working Party, and the Cord Blood Committee of the Cellular Therapy and Immunobiology Working Party of the European Group for Blood and Marrow Transplantation
Source: J Hematol Oncol. 2015 Oct 6;8:107. doi: 10.1186/s13045-015-0207-4 (PMC4594748; doi:10.1186/s13045-015-0207-4)
Supplement: Additional file 1: — List of institutions reporting data in this study. [file 13045_2015_207_MOESM1_ESM.docx]

**Supplemental material : list of institutions reporting data in this study**

Hospital Universitario La Fe, Servicio de Hematologia, Valencia, Spain; CHU Bordeaux, Hôpital Haut-leveque, Pessac, France; Ospedale San Martino, Department of Haematology II, Genova, Italy; Hopital Saint Antoine, Department of Hematology, Paris, France; Azienda Ospedaliera Papa Giovanni XXIII, Hematology and Bone Marrow Transplant Unit, Bergamo, Italy; Bologna University, S.Orsola-Malpighi Hospital, Institute of Hematology & Medical, Oncology L & A Seràgnoli, Bologna, Italy; Ospedale di Careggi, BMT Unit Department of Hematology, Firenze, Italy; Hospital Santa Creu i Sant Pau, Hematology Department, Barcelona, Spain; Hôpital HURIEZ, UAM allo-CSH, CHRU, Lille, France; ICO-Hospital Universitari Germans Trias i Pujol, Cattedra e Servizio di Ematologia, Barcelona, Spain; Medical University Graz, LKH - University Hospital Graz, Division of Haematology, Graz, Austria; Clinica Puerta de Hierro, Servicio de Hematologia y Hemoterapia, Madrid, Spain; Hospital Clinic, Institute of Hematology & Oncology, Dept. of Hematology, Barcelona, Spain; ¨Tor Vergata¨ University of Rome, Stem Cell Transplant Unit, Policlinico Universitario Tor Vergata, Rome, Italy; Az. Ospedaliera S. Croce e Carle, Division of Hematology, Cuneo, Italy; Hospital Gregorio Marañón, Sección de Trasplante de Medula Osea, Madrid, Spain; Programme de Transplantation&Therapie Cellulaire, Centre de Recherche en Cancérologie de Marseille, Institut Paoli Calmettes, Marseille, France; CHRU St. Etienne, Hopital Nord, Service d`Hematologie Clinique, Saint_Etienne, France; CHU Nice - Hôpital de l`ARCHET I, Hematologie Clinique, Nice, France; Hopital La Miletrie, Head of the Bone Marrow TransplantUnit, Clinical Hematology, Poitiers, France; Hopital A. Michallon, Department of Hematology, Grenoble, France; Centre Hospitalier Lyon Sud, Service Hematologie, Lyon, France; Hôpital Henri Mondor, Sve d` Hematologie, Creteil, France; Hospital Clínico Universitario, Servicio de Hematologia y Oncologia, Valencia, Spain; CHRU, Service des Maladies du Sang, Angers, France; University of Liege, Dept. of Hematology, CHU Sart-Tilman, Liege, Belgium; Royal Marsden Hospital, Leukaemia Myeloma Units, London, United Kingdom; Hospital Clínico, Servicio de Hematología, Salamanca, Spain; Chaim Sheba Medical Center, Chaim Sheba Medical Center, Dept. of Bone Marrow Transplantation, Tel-Hashomer, Israel; Hospital Universitario Virgen del Rocío, Servicio de Hematologia y Hemoterapia, Servicio Andaluz de Salud, Sevilla, Spain; Azienda Ospedali Riuniti di Ancona, Department of Hematology, Ancona University, Ancona, Italy; Karolinska University Hospital, Dept. of Hematology, Stockholm, Sweden; Hosp. Reina Sofia, Córdoba Hospital, Department of Hematology, Córdoba, Spain; Istituto Clinico Humanitas, Transplantation Unit, Department of Oncology and Haematology, Milano, Italy; Ospedale V. Cervello, Div. di Ematologia e Unità Trapianti, Palermo, Italy; Elisabethinen-Hospital, I. Internal Department, Linz, Austria; Hospital Morales Meseguer, Unidad de Trasplante de Médula Osea, Serv de Hemat, Murcia, Spain; Centre Henri Becquerel, Hematology, Rouen, France; USD Trapianti di Midollo, Adulti, Universita di Brescia, Brescia, Italy; S.S.C.V.D Trapianto di Cellule Staminali, Torino, Italy; Universite Paris IV, Hopital la Pitié-Salpêtrière, Hematologie Clinique, Paris, France; Beilinson Hospital, Hematology and BMT Department, Petach-Tikva, Israel; King Hussein Cancer Centre, Amman, Jordan; Ankara University Faculty of Medicine, Dept. of Hematology, Adult Stem Cell Transplantation Unit, Dikimevi, Ankara, Turkey; Hopital d`Enfants, Unité de Transplantation Médullaire, Service de Méd. Infantile, Vandoeuvre_Les_Nancy, France; University Hospital Gasthuisberg, Dept. of Hematology, Leuven, Belgium; Rikshospitalet, Department of Medicine, The National Hospital, Oslo, Norway; Hospital U. Marqués de Valdecilla, Servicio de Hematología-Hemoterapia, Santander, Spain; Hadassah University Hospital, Dept. of Bone Marrow Transplantation, Jerusalem, Israel; BMT unit, Clinica Ematologica, Fondazione IRCCS Policlinico San Matteo, Pavia, Italy; Sahlgrenska University Hospital, Center for Hematopoietic Cell Transplantation, Hematology Section, Goeteborg, Sweden; Department of Haematology, University Hospital of Wales, Cardiff, United Kingdom; Onco-Ematologia Pediatrica, Centro Trapianti Cellule Staminali, Ospedale Infantile Regina Margherita, Torino, Italy; U.O.S.A Centro Trapianti e Terapia Cellulare, Azienda Ospedaliera Universitaria Senese, Policlinico S.Maria alle Scotte, Siena, Italy; King Faisal Specialist Hospital & Research Centre, Oncology (Section of Adult Haematolgy/BMT), Riyadh, Saudi Arabia; Klinikum Grosshadern, Med. Klinik III, Munich, Germany; Ospedale San Gerardo, Clinica Ematologica dell`Universita Milano-Biocca, Monza, Italy; Hospital Univ. Virgen de las Nieves, Servicio de Hematología, Granada, Spain; Hospital Regional de Málaga, Servicio de Hematología, Málaga, Spain; Hospital Vall d`Hebron, Unidad de Adultos, Barcelona, Spain; Evangelismos Hospital, Division of Hematology, BMT Unit, Athens, Greece; Institut Universitaire du Cancer Toulouse, Oncopole, Toulouse, France; Hospital Universitario Central de Asturias, Avenida de Roma S/N, Oviedo, Spain; Hôpital Percy, Hematology Department, Clamart, France; Gustave Roussy, institut de cancérologie, BMT Service, Division of Hematology, Department of Medical Oncology, Villejuif, France; Azienda Policlinico Vittorio Emanuele, Programma di Trapianto Emopoietico Misto e Metropolitano Di Catania, Ospedale Ferrarotto, Catania, Italy; Ospedale San Raffaele s.r.l., Haematology and BMT, Milano, Italy; CHRU Limoges, Service d`Hématologie Clinique, Limoges, France; Leiden University Hospital, BMT Centre Leiden, Leiden, Netherlands, The; Hopital St. Louis, Dept.of Hematology - BMT, Paris, France; University Hospital, Clinic of Hematology, Zürich, Switzerland; Univ.`La Sapienza`, Dip. Biotecnologie Cellulari ed Ematologia, Rome, Italy; Cliniques Universitaires St. Luc, Dept. of Haematology, Brussels, Belgium; CHU CAEN, Institut d’hématologie de Basse-Normandie, Caen, France; CHU Nantes, Dept. D`Hematologie, Nantes, France; Fondazione IRCCS Ca’ Granda Ospedale Maggiore Policlinico, IRCCS, Milano, Italy; University Hospital, Dept. of Medicine, Uppsala, Sweden; Ospedale S. Camillo-Forlanini, Dept. of Hematology and BMT, Rome, Italy; Rambam Medical Center, Dept. of Hematology & BMT, Haifa, Israel; Bristol Royal Hospital for Children, Dept. of Paediatric Oncology/BMT, Bristol, United Kingdom; Heinrich Heine Universität, Klinik für Hämat,Onkol,Klin.Immun., Düsseldorf, Germany; Universitätsklinikum Jena, Klinik für Innere Medizin II, Jena, Germany; Dept. Haematology and Stem Cell Transplant, St. István and St. László Hospital, Budapest, Hungary; Wellington Hospital, Wellington Regional Oncology Unit, Wellington, New Zealand; Manchester Royal Infirmary, Clinica Haematology Department, Manchester, United Kingdom; Hospital Ampang, Jalan Mewah Utara, Ampang, Malaysia; Policlinico G.B. Rossi, Verona, Italy; C.H.R.U de Brest, Brest, France; Leicester Royal Infirmary, Department of Haematology, Leicester, United Kingdom; Nottingham City Hospital, Hucknall Road, Nottingham, United Kingdom; Hospital Universitario La Paz, Hematologia-Oncologia, Madrid, Spain; ICO – Hospital Duran i Reynals, Barcelona, Spain; GKT School of Medicine, Dept. of Haematological Medicine, London, United Kingdom; Canterbury Health Laboratories, Christchurch, New Zealand; Universitaetsklinikum Dresden, Medizinische Klinik und Poliklinik I, Dresden, Germany; H SS. Antonio e Biagio, Haematology Department, Alessandria, Italy.
